# Cerebral Ischemia due to Aortic Arch Thrombosis Secondary to Iron Deficiency Anemia

**DOI:** 10.1155/2019/8647126

**Published:** 2019-07-18

**Authors:** Taha Nisar, Rajanigandhi Hanumanthu

**Affiliations:** Department of Neurology, Rutgers New Jersey Medical School, Newark, New Jersey, USA

## Abstract

Thrombocytosis, hypercoagulable state, and hypoxia secondary to anemia are some of the mechanisms that are thought to cause strokes in patients with iron deficiency anemia (IDA). Several cases of middle-aged females with IDA who had embolic strokes due to aortic arch thrombosis have been reported. Majority of the cases were treated with anticoagulation. We report another case of embolic strokes in a patient with IDA treated with anticoagulation and iron replacement without recurrence of further episodes. We concluded that embolic phenomenon in middle-aged females with IDA warrants transesophageal echocardiogram with an evaluation of aortic arch.

## 1. Introduction

Iron deficiency anemia (IDA) is a cause of secondary thrombocytosis and commonly affects women of reproductive age [1]. The exact mechanism of thrombocytosis in the setting of IDA is unclear, and several theories exist [1, 2]. Several cases of middle-aged females with IDA who had embolic strokes due to aortic arch thrombosis have been reported [2–4]. We present another interesting case of an embolic phenomenon in a middle-aged female with IDA, who had a thrombus visualized in the aortic arch on evaluation with a transesophageal echocardiogram (TEE), without evidence of atherosclerotic disease. Our patient was treated with anticoagulation and iron replacement without recurrence of further episodes.

## 2. Case Report

**Figure 1:**
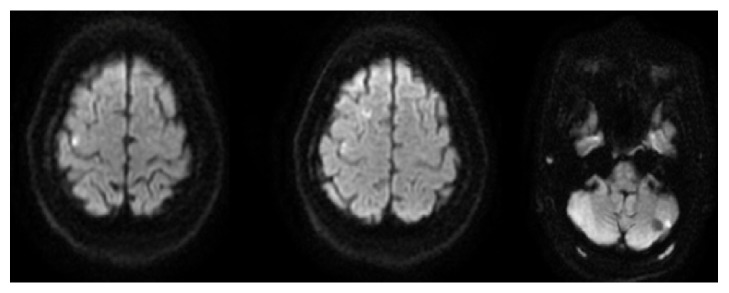
MRI of the brain without contrast showing small foci of diffusion restriction involving the right frontal lobe and left cerebellar hemisphere consistent with areas of acute infarctions.* Abbreviation.* MRI: magnetic resonance imaging.

**Figure 2:**
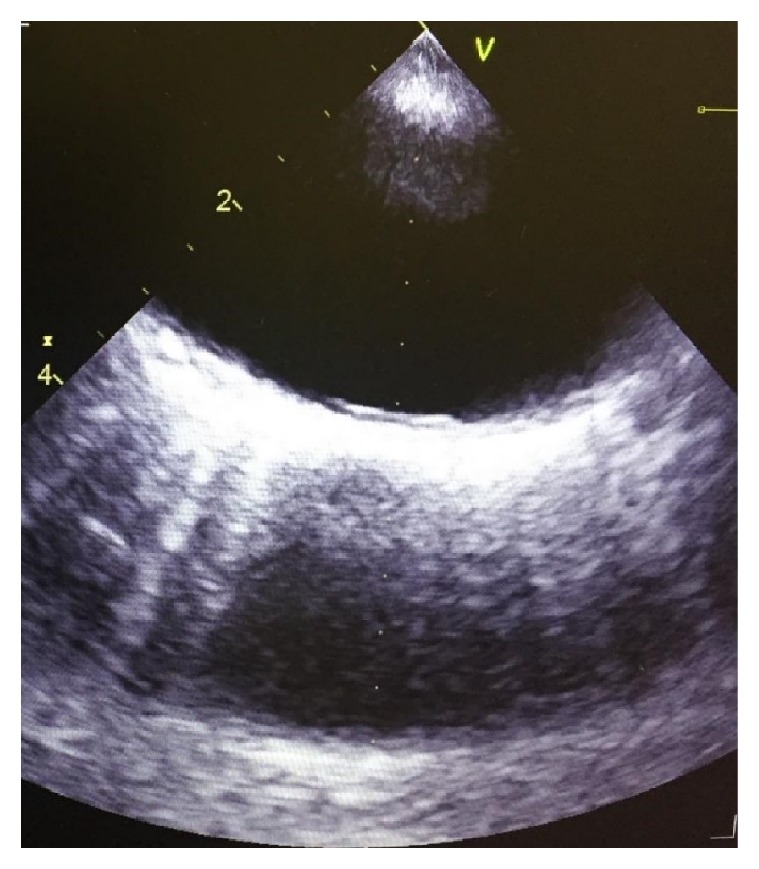
TEE demonstrating an echogenic density in the aortic arch, consistent with thrombus.* Abbreviation.* TEE: transesophageal echocardiogram.

**Figure 3:**
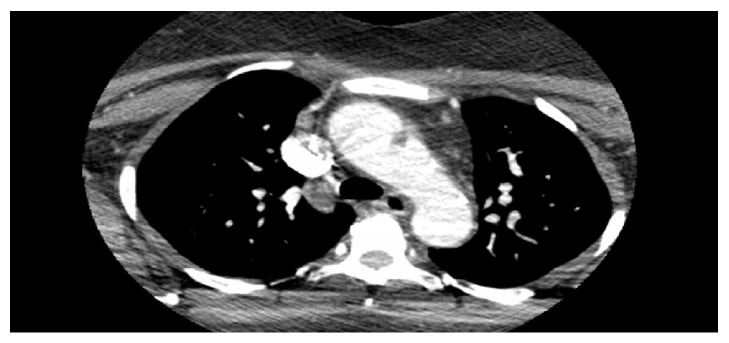
CT Angiogram of head and neck showing a 1.3 cm filling defect in the proximal aortic arch abutting the lateral wall of the aortic arch, consistent with thrombus.* Abbreviation.* CT: computed tomography.

48-year-old African American woman with a history of menorrhagia presented to the emergency department with a complaint of a transient episode of weakness and numbness in her left arm and leg, lasting only 2 minutes. On presentation, her vitals were as follows: blood pressure: 151/81 mm Hg, heart rate: 108 beats/minute, temperature: 98.2 degrees Fahrenheit, and respiratory rate: 20. Upon presentation to the emergency department, her neurological examination was nonfocal. Her labs were suggestive of iron deficiency anemia (IDA) (Hb: 6.0 g/dl, MCV: 56.2 *μ*m3, platelets: 555 x1000 *μ*l, serum iron: 11 *μ*l/dl, total iron binding capacity (TIBC): 425 *μ*g/dl, ferritin: 4ng/ml, hemoglobin electrophoresis: 100% hemoglobin A, vitamin B 12, and folate were normal). She received a unit of packed red blood cells. Magnetic resonance imaging (MRI) of the brain showed acute infarctions in the right frontal lobe and left cerebellar hemisphere, as shown in Figure 1. There was a suspicion for embolic etiology given the bilateral location of the stroke, and the patient was started on anticoagulation. Transesophageal echocardiography (TEE) showed 0.9 X 0.7[2–4] cm echogenic density in the aortic arch, as shown in Figure 2. Computed tomography (CT) angiogram did not show any evidence of atherosclerotic disease but showed a 1.3 cm filling defect in the aortic arch, consistent with thrombus as shown in Figure 3. Repeat MRI of the brain 4 days later did not show any new strokes, and her neurological examination remained stable.

**Figure 4:**
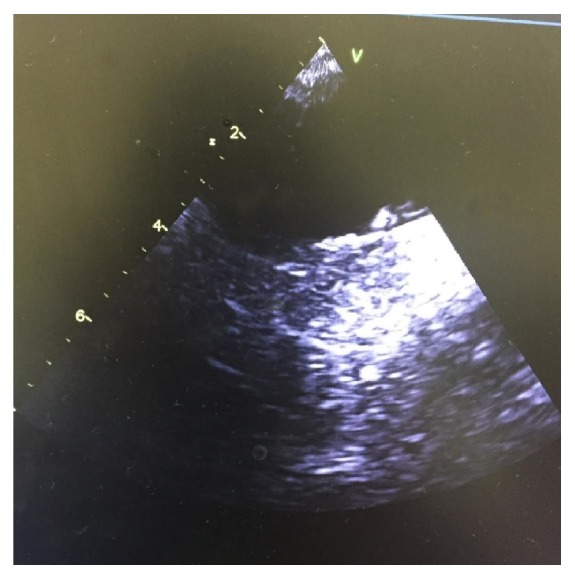
Follow-up TEE performed four months later demonstrating that prior echogenic mass in the aortic arch had decreased in size.* Abbreviation.* TEE: transesophageal echocardiogram.

Further workup showed beta-2 glycoprotein IgM & IgG, anticardiolipin IgM & IgG, Sjogren Anti-SSA & Anti-SSB, antiextractable nuclear AG (RNP, Smith), C3, C4 complements, protein C, and factor II mutation within normal limits. MTHFR mutation was heterozygous in the A gene only, while homocysteine was normal. The patient was discharged on warfarin, aspirin, and iron supplementation. Repeat TEE at four months showed that the prior echogenic mass in the aortic arch had decreased in size, as shown in Figure 4. She did not report further clinical events in eight-month follow-up and remained on warfarin and iron repletion.

## 3. Discussion

In 1990, Tunick et al. became the first to describe 3 cases of embolic strokes attributed to freely mobile aortic arch plaques that were visualized on a TEE. All the three patients reported had a severe atherosclerotic disease [5]. Aortic arch thrombosis is usually described in the context of severe atherosclerotic disease. However, in 1997, Laperche et al. published a case series of patients with recent arterial embolism due to mobile aortic arch thrombosis without diffuse aortic atherosclerotic debris [6]. Our patient had IDA and aortic arch thrombosis in the absence of atherosclerotic disease. However, the absence of macroscopic or iconographic evidence of atherosclerotic lesions does not eliminate the possibility of microscopic atherosclerotic lesions. Literature review shows several cases with a presentation similar to our patient, as shown in Table 1 [2–4]. The majority of the cases were treated with anticoagulation (heparin and warfarin), while one patient was treated with total arch replacement. Furthermore, several cases of IDA's association with carotid thrombosis and embolic strokes have also been reported [7].

Secondary (reactive) thrombocytosis makes up around 88% of all causes of thrombocytosis [1]. IDA is a cause of secondary thrombocytosis [1]. The exact mechanism of thrombocytosis in IDA is unclear, and several theories exist. Thrombocytosis, hypercoagulability, and hypoxia secondary to anemia are some of the mechanisms which are thought to trigger thrombosis and cause strokes in patients with IDA. It is thought that thrombopoietin is a glycoprotein hormone synthesized in the liver and kidneys which regulates the production of platelets by regulating megakaryocyte differentiation and proliferation. Megakaryocytes and platelets have c-Mpl receptors on the surface. Unbound thrombopoietin present in plasma interacts with c-Mpl receptors, promoting the proliferation of megakaryocytes. A fall in platelet count leads to an increase in free unbound thrombopoietin which in turn interacts with c-Mpl receptors on megakaryocytes, stimulating their maturation. Conversely, an increase in platelet count decreases free unbound thrombopoietin with a decrease in its interaction between c-Mpl receptors on megakaryocytes, decreasing maturation. IL-6 is thought to upregulate thrombopoietin messenger RNA in the liver in acute phase response. Thus interactions between IL-6, thrombopoietin, and c-Mpl receptors on megakaryocytes and platelets regulates platelet proliferation and maturation [1].

IDA is also thought to cause hypercoagulability by altering the blood flow dynamics in major vessels. If we take into account Reynolds number= R vD/N (where R is the radius of the vessel, v is the flow velocity, d is the density of blood, and n is the viscosity of blood), we can conclude that increased turbulent flow is caused in IDA. Reynolds number is elevated due to an increase in blood flow velocity (v) and a decrease in blood viscosity (n). The Virchow's triad of blood stasis, endothelial injury, and hypercoagulability can explain thrombosis in this scenario [7].

Anemia is associated with higher stroke mortality [8]. While anticoagulation is the mainstay of aortic arch thrombosis, surgical options can also be considered due to their safety profile in younger patients but remain contentious, as aortic arch surgery is itself a risk factor for embolic strokes [9–12].

## 4. Conclusion

An embolic phenomenon in middle-aged females with IDA warrants a TEE with an evaluation of aortic arch. If an aortic arch thrombosis is discovered, the patient should be anticoagulated.

## Figures and Tables

**Table 1:** Case Reports of patients with embolic strokes in the setting of aortic arch thrombosis in the setting of iron deficiency anemia.

Case reports	Demo- graphics	Past Medical History	Location of stroke	Aortic Imaging	Labs	Anemia Workup	Treatment Given	Outcome
Yakushiji et al (2005)[4]	50-year-old woman	Occasional hematochezia for 2 months before the admission Anorexia since the age of 17 years	Left MCA M1	*TEE:* mobile mass (18 x 8 mm) in the aortic arch No atherosclerotic changes on TEE	Hb (g/dl): 5.5 (12-16.5) MCV (microm3): 62 (85-100) Platelet (x1000 microl): 420 (150-350) Iron(microl/dl): 16 (43-172) Ferritin (microl/dl): 21 (0-429) Protein S activity (%): 36 (65-105) Reticulocytes (%): 10 (5-20)	Internal hemorrhoids	Blood transfusion Heparin drip	On day 19 TEE & Cardiac MRI showed resolution of aortic arch clot Switched to ASA from warfarin at 1 year On 2-year follow-up, there was no recurrence of IDA or stroke

Yakushiji et al (2005)[4]	41-year-old woman	Epimenorrhagia since the age of 20 years.	Left ACA, Left MCA, Bilateral Cerebellum	*TEE:* mobile mass (5 x 10 mm) in the aortic arch. No atherosclerotic changes on TEE	Hb (g/dl): 7.9 (12-16.5) MCV (microm3): 61 (85-100) Platelet (x1000 microl): 360 (150-350) Iron (microl/dl): 9 (43-172) Ferritin (microl/dl): 2 (0-429) Protein S activity (%): 54 (65-105) Reticulocytes (%): 23 (5-20)	Adenomyosis uteri.	Heparin drip	On day 7 TEE & Cardiac MRI showed resolution of aortic arch clot On 6-month follow-up, there was no recurrence of IDA or stroke Switched to ASA from warfarin at 1 year

Bukharovich et al (2012)[2]	49-year-old Caucasian woman	Menorrhagia	Right sided multiple infarcts	*TEE:* mobile mass (10 x 6 mm) in the aortic arch. No atherosclerotic changes on TEE	Hb (g/dl): 8.4 (12-16.5) MCV (microm3): 68 (85-100) Platelet (x1000 microl): 567 (150-350) Iron(microl/dl): 20 (43-172) Ferritin (microl/dl): 15 (0-429)	No GI source Negative gynecological evaluation	Heparin drip Oral Iron	Discharged after 10 days on Warfarin

Ishii et al (2017) [3]	41-year-old woman	Affective Disorder	Left M 2	*TEE:* mobile mass (10 x 6 mm) in the aortic arch. No atherosclerotic changes on TEE	Hb (g/dl): 7.5 (12-16.5)	Whole-body MRI: Uterine fibroids and endometrial cysts	Total arch replacement	

*Abbreviations:* MCA: middle cerebral artery; ACA: anterior cerebral artery; M1: the proximal segment of middle cerebral artery before bifurcation; M2: a segment of the middle cerebral artery after bifurcation before further subdivision; Hb: hemoglobin; TTE: transesophageal echocardiogram; MRI: magnetic resonance imaging; MCV: mean corpuscular volume.
